# Estimating Umbilical Venous Catheter Insertion Depth in Newborns Using Weight or Body Measurements: A Multicenter Randomized Clinical Trial

**DOI:** 10.7759/cureus.90276

**Published:** 2025-08-17

**Authors:** Fawzy K Abdelhamid, Abdulrahman Al Matery, Bader Z Mahmoud, Tagwa B Eltayeb, Mohammed Almasoud, Emad A Alhulaimi, Mohammed H Alqahtani, Mohammed Y Al-Hindi

**Affiliations:** 1 Neonatology, Al-Yamamah Hospital, Al Riyadh, SAU; 2 Neonatology, Health Sciences Centre Winnipeg, Winnipeg, CAN; 3 Neonatology, King Fahad Medical City, Al Riyadh, SAU; 4 Neonatology, Alyamah Hospital, Al Riyadh, SAU; 5 Collage of Medicine, King Saud Bin Abdulaziz University for Health Sciences College of Medicine, Jeddah, SAU; 6 Research and Development, King Abdullah International Medical Research Center, Jeddah, SAU; 7 Pediatrics, King Abdulaziz Medical City-Wr, Ministry of National Gaurd, Jeddah, SAU

**Keywords:** appropriate for gestational age (aga), birth weight-based formula, birth weight (bw), catheter placement, clinical trial, gupta method, neonatal intensive care unit, small for gestational age (sga), umbilical venous catheter (uvc)

## Abstract

Introduction

The current methods for estimating umbilical venous catheter (UVC) insertion depth, including the umbilical to nipple (Gupta) and the birth weight-based (modified Shukla) formula, have varying accuracy rates.

Objectives

To compare the accuracy of UVC insertion length using the birth weight-based formula versus the surface measurement formula in determining the optimal UVC tip position.

Methods

A multicenter randomized clinical trial was conducted across three centers, including level III neonatal intensive care units (NICUs) in the Second Cluster in Riyadh (Al-Yamama Hospital and King Fahad Medical City), as well as King Salman Armed Forces Hospital in Tabuk City, Northwestern Region, Saudi Arabia. Neonates requiring UVC insertion during their NICU admission were randomly assigned to one of two formulas: (1) the umbilicus to nipple distance in centimeters minus 1 (UN - 1) or (2) the modified Shukla weight-based formula in centimeters (3×birth weight in kg+9, divided by 2) to estimate the pre-insertion UVC depth and to determine the UVC tip position anteroposterior and lateral thoracoabdominal radiographs were taken and reviewed by a neonatologist, who was blinded to the group assignments. Data analysis was conducted using appropriate statistical methods, ensuring adherence to ethical standards.

Results

A total of 158 infants were analyzed, with 88 in the Gupta group and 70 in the modified Shukla group. The majority of UVC insertions (n=156, 98.7%) were performed for intravenous fluids, total parenteral nutrition (TPN), and antibiotics, while only two cases (n=2, 1.3%) involved blood exchange transfusion. The demographic and clinical data of the two groups were comparable. The Gupta method demonstrated a significantly higher rate of correct catheter placements (n=55, 62.5%) compared to the modified Shukla method (n=32, 45.7%; p=0.02). The Gupta method was particularly effective in the SGA group, with 70.0% (n=21) correct placements, compared to 42.9% (n=9) in the modified Shukla group (p=0.04). No significant differences in catheter advancement rates or fix-on measurements were observed between the two methods (p=0.59 and p=0.45, respectively). The modified Shukla group had a significantly abnormal catheter low tip position (n=23, 32.9%) compared to the Gupta group (n=13, 14.8%; p=0.02).

Conclusions

The Gupta method provides more accurate UVC placement, especially in SGA neonates, compared to the modified Shukla method. Both methods showed similar catheter advancement success and insertion depth, suggesting that the Gupta method may be a more reliable approach for UVC insertion in neonates. Further studies with larger sample sizes and advanced imaging techniques are needed to confirm these findings and assess long-term outcomes.

## Introduction

Umbilical vessel catheterization has been a critical procedure in neonatal care for over 60 years, providing essential venous access in newborns, particularly those who are preterm or have severe morbidity [1,2]. This procedure facilitates the administration of fluids, parenteral nutrition, blood products, antibiotics, and other vital interventions in neonatal intensive care units (NICUs) [2]. Despite its widespread use, the accurate placement of the umbilical venous catheter (UVC) remains a challenge, with the ideal position of the UVC tip being at the junction of the inferior vena cava and the right atrium to minimize complications [3]. Improper positioning, particularly when the catheter tip lies within the heart, can lead to severe complications such as pericardial effusions, cardiac tamponade, and thrombus formation [4].

Several methods have been proposed to estimate the appropriate insertion depth of UVCs, including the umbilical to nipple minus one (Gupta) method, the Shukla-Ferrara method, and various modified formulas. However, no single method has been universally accepted, and the selection of the most accurate method is crucial to reducing the risk of complications [5,6]. In the NICUs at Second Cluster in Al Riyadh (Al Yamama Hospital and King Fahad Medical City) and King Salman Armed Forces Hospital in the Northwestern Region, Tabuk City, Saudi Arabia, the most commonly used formula for estimating UVC insertion length is based on birth weight (3×birth weight (kg)+9)/2. However, this formula has shown an accuracy rate of only 57% in a retrospective study, highlighting the need for more reliable methods [7].

In contrast, another study has suggested that a formula based on the distance from the base of the umbilicus to the nipple minus 1 cm (UN - 1 cm) offers a significantly higher accuracy rate of 94% for UVC placement [8]. Despite these findings, there is a lack of comprehensive audits comparing the accuracy rates of these methods within our units. The endpoint measurement of the UVC tip, typically confirmed via anteroposterior and lateral X-ray [9,10], remains the standard practice in our units. Given the critical importance of precise catheter placement in neonatal care, this study aims to evaluate the accuracy of UVC insertion depth estimation using the umbilical to nipple minus one (Gupta method) compared with the modified Shukla formula (3*weight+9)/2, based on birth weight.

## Materials and methods

This randomized clinical trial was conducted in the NICUs of the Second Cluster in Al Riyadh (Al Yamama Hospital and King Fahad Medical City) and King Salman Armed Forces Hospital in the Northwestern Region, Tabuk City, Saudi Arabia. The primary objective was to compare two formulas for estimating the insertion depth of UVCs in newborns who required this procedure as part of their intensive care. The study population included neonates up to 14 days of age, of both sexes, who were admitted to the NICUs in the studied hospitals and required UVC insertion during their stay. The inclusion criteria were broad, encompassing all neonates who required UVC insertion, while the exclusion criteria were specific, ruling out those with medical contraindications for UVC insertion, such as lower limb ischemia, omphalitis, umbilical bleeding, confirmed intravascular thrombosis, disseminated intravascular coagulation, hydrops fetalis, abdominal wall defects, congenital diaphragmatic hernia, major congenital heart disease, and those whose parents declined participation or failed to insert UVC to desired measurement. Additionally, neonates who required UVC insertion beyond two weeks of life were excluded.

**Figure 1:**
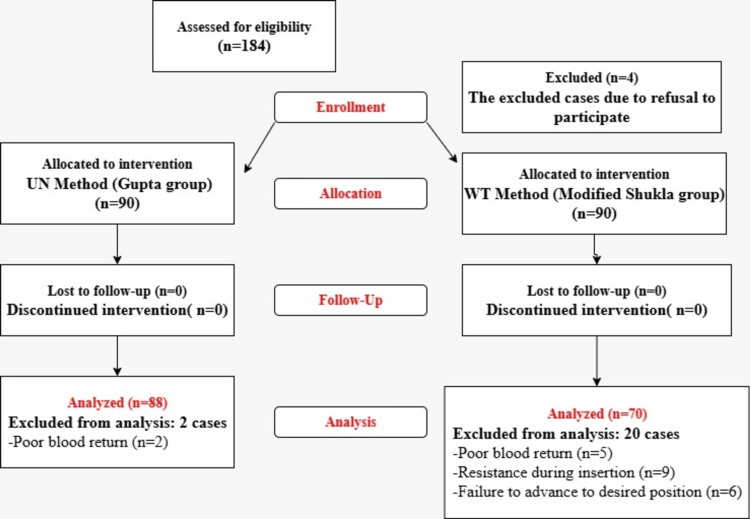
Flow Diagram of Studied Neonates

Neonates who met the eligibility criteria were randomly assigned to one of two groups. Group 1 had their UVC insertion depth estimated using the umbilicus to nipple distance (UN) minus 1 cm formula (Gupta method), while Group 2 used the birth weight formula (3*wt+9)/2 (modified Shukla). Additionally, both groups will add the measurement of the umbilical stump in centimeters to their respective estimates to refine the final insertion depth. Randomization was achieved using a computer-generated sequence, with allocation in blocks of four. The neonates were randomized in a 1:1 ratio. In the present study, 184 infants met the inclusion criteria. Of these, four were excluded due to a refusal to participate. The remaining 180 infants were randomly assigned to one of two intervention groups (the Gupta group or the modified Shukla group) in a 1:1 ratio. All infants in both groups received the allocated intervention, with no cases lost to follow-up or discontinuation of the intervention. However, two infants from the Gupta group were excluded due to poor blood return, and 20 infants from the modified Shukla group were excluded for the following reasons: poor blood return (n=5), resistance during insertion (n=9), or failure to advance to the desired position (n=6). As a result, the final analysis included 158 infants: 88 from the Gupta group and 70 from the modified Shukla group. The demographic and clinical data of the included and excluded infants were comparable across both groups. All infants in both groups received the allocated intervention, with no cases lost to follow-up or discontinuation of the intervention. A flow chart outlining the studied cases is presented in Figure 1.

The two groups were then stratified based on birth weight, dividing the neonates into two groups: those weighing less than 1500 grams and those weighing 1500 grams or more. As a result, the study included four groups: less than 1.5 kg - UN method, less than 1.5 kg - weight method, 1.5 kg or more - UN method, and 1.5 kg or more - weight method. 

UVC insertion was performed under sterile conditions according to the unit protocol by a senior NICU physician. For term and preterm deliveries, written informed consent was obtained from a parent before enrollment in the study. In emergency cases, where the neonate required immediate UVC insertion upon admission to the NICU, a waiver of informed consent was applied. In such instances, parents were informed as soon as possible about their infant’s participation in the study, provided with written information, and asked for their consent to include their infant’s data in the analysis, with the option to give consent via telephone if they were unable to be present in person.

After UVC insertion, anteroposterior and lateral thoracoabdominal radiographs were performed to verify the position of the UVC tip, ensuring it was located at the correct anatomical level on the anteroposterior and lateral radiograph, and reviewed by a certain group of neonatologists, who were blinded to the group assignments. The ideal position for the tip is at the upper border of the 9th thoracic vertebra or the lower border of the 10th thoracic vertebra. On the lateral radiograph, the tip should be within 0.5 cm above or below the diaphragm, which typically aligns with the junction of the inferior vena cava (IVC) and the right atrium for correct UVC placement. The case was included in the study if the tip was positioned above or below the specified range, provided there was good blood return, no resistance during insertion, and the catheter advanced to the desired measurement. Conversely, regardless of whether the tip was in the atrium, portal vein, ductus venosus, or any other abnormal position, the case was excluded if there was poor blood return, resistance during insertion, or failure to advance to the desired position. Safety measures during the procedure included continuous ECG monitoring to detect any arrhythmias or signs of general deterioration. The insertion site was regularly assessed for signs of infection, such as erythema, tenderness, or swelling, and the limb’s circulation was checked hourly. Any adverse findings were documented and promptly reported to the medical team. The medical team also reviewed the need for continued UVC placement on a daily basis.

The primary outcome measure for this study was the proportion of UVCs correctly positioned at the optimal catheter tip location using each method. Secondary outcomes included the number of UVC readjustments required and a comparison of UVC tip position between the two formulas based on the neonate’s growth status at birth (appropriate for gestational age (AGA), small for gestational age (SGA), or large for gestational age (LGA)), and if there are any complications.

Data analysis was conducted using the IBM SPSS Statistics (version 22.0, IBM Corp., Armonk, NY). Continuous variables were reported as mean±standard deviation, while categorical data were presented as frequencies and percentages. Pearson’s Chi-square and Fisher exact tests were used to compare categorical variables across groups as appropriate, and independent t-test, was used to compare continuous variables. A p-value less than 0.05 was considered statistically significant. An intention-to-treat analysis was performed, where each case was analyzed within its assigned group, excluding those with missing data and those who discontinued the interventions. Ethical approval for this study was obtained from the research committee of the Riyadh Health of Second Cluster (AL Yamama Hospital and King Fahad Medical City), King Salman Armed Forces Hospital in Northwestern Region, and Tabuk City, and registration number in ClinicalTrials.gov was NCT07045506. The study adhered to the ethical principles outlined in the Declaration of Helsinki, ensuring that parents were fully informed about the study and their consent was obtained.

## Results

A total of 158 infants were analyzed (88 in the Gupta group and 70 in the modified Shukla group). The vast majority of UVC insertions (n=156, 98.7%) were performed for administering intravenous fluids, total parenteral nutrition (TPN), and medication as antibiotics, with only two cases (n=2, 1.3%) involving blood exchange transfusion. Furthermore, the majority of UVCs (n=155, 98%) were inserted on the day of birth, with the mean duration of umbilical venous catheter placement being 6.9±3.2 days (range, 1-14 days). There was no statistically significant difference between the groups in this regard (p=0.62). Table 1 presents the demographic and clinical characteristics of the studied neonates categorized by the Gupta and modified Shukla's groups. The gender distribution is similar across both groups, with 48.9% (n=43) female infants and 51.1% (n=45) male infants in the Gupta group, and 54.3% (n=38) female infants and 45.7% (n=32) male infants in the modified Shukla group (p=0.50). The mode of delivery shows a higher percentage of vaginal births in the Gupta group (n=61, 69.3%) compared to the modified Shukla group (n=37, 53.0%), while cesarean section and assisted breech deliveries are more common in the modified Shukla group, but the difference is not statistically significant (p=0.10). The birth weight, birth height, head circumference, and gestational age are also similar between the two groups (gestational age (weeks); mean±SD of 31.0±4.8 for Gupta group and 30.4±4.5 for modified Shukla’s group) with no significant differences found (p=0.16, p=0.35, p=0.30, and p=0.37, respectively). In terms of gestational age categories, most neonates in both groups were preterm, with 80.7% (n=71) in the Gupta group and 85.7% (n=60) in the modified Shukla group, but the difference is not significant (p=0.40). Weight by gestational age shows that the majority of neonates in both groups are appropriate for gestational age (AGA), with no significant differences in the distribution between the groups (p=0.60). The reasons for hospitalization, including conditions such as respiratory distress syndrome (RDS), cardiopulmonary resuscitation (CPR), very low birth weight (VLBW), and encephalopathy, also do not show significant differences between the two groups (p=0.89). Overall, the two groups showed similar characteristics in the variables studied.

**Table 1:** Demographic and clinical characteristics of the studied neonates by groups *Significant. AGA: appropriate for gestational age, SGA: small for gestational age, LGA: large for gestational age, CPR: cardiopulmonary resuscitation, RDS: Respiratory distress syndrome, PT: Preterm, VLBW: Very low birth weight, XVLBW: Extremely very low birth weight. ^a^Chi-square test, ^b ^Independent t-test was used.

Characteristics	Gupta group (n=88), n (%)	Modified Shukla’s group (n=70), n (%)	Test value	P value
Gender^a^				
Female	43 (48.9)	38 (54.3)	0.46	0.50
Male	45 (51.1)	32 (45.7)		
Mode of delivery				
Vaginal	61 (69.3)	37 (53.0)	4.61	0.10
C/S	23 (26.2)	29 (41.3)		
Assisted breech	4 (4.5)	4 (5.7)		
Type of delivery				
Single	67 (76.1)	57 (81.4)	0.65	0.42
Twins	21 (23.9)	13 (18.6)		
Birth weight (gm); Mean±SD^b^	1569.1±921.3	1378.1±767.0	1.42	0.16
Birth height (cm); Mean±SD^b^	39.5±6.6	38.6±5.4	0.90	0.35
Head circumference (cm); Mean±SD^b^	28.7±3.9	28.1±3.3	1.03	0.30
Gestational age (weeks); Mean±SD	31.0±4.8	30.4±4.5	0.90	0.37
Gestational age categories				
<37 weeks	71 (80.7)	60 (85.7)	0.70	0.40
≥37 weeks	17 (19.3)	10 (14.3)		
Weight by gestational age				
AGA	55 (62.5)	48 (68.6)	0.97	0.60
LGA	3 (4.3)	1 1.4)		
SGA	30 (34.1)	21 (30.0)		
Reasons for hospitalization				
RDS and preterm	30 (34.1)	27 (39.0)	1.10	0.89
CPR	5 (5.7)	3 (4.2)		
VLBW and XVLBW	5 (5.7)	2 (2.9)		
Encephalopathy	3 (3.4)	2 (2.9)		
Combination including (PT, RDS, VLBW, LBW, TOF)	45 (51.1)	36 (51.0)		

The data presented in Table 2 compares the UVC tip positions between the Gupta and modified Shukla groups, as determined by X-ray. In the Gupta group, 62.5% (n=88) of the catheters were placed in the correct position, while 45.7% (n=70) of the catheters in the modified Shukla group achieved correct placement. This difference was found to be statistically significant with a p-value of 0.02. The high position was observed in 22.7% (n=20) of the Gupta group and 21.4% (n=15) of the modified Shukla group, showing minimal difference between the two groups. However, a notable difference was observed in the placement of the catheter in the low position, with 14.8% (n=13) of the Gupta group versus 32.9% (n=23) of the modified Shukla group having the catheter tip in the low position. The statistically significant finding (p=0.02) highlights that the Gupta group had a higher percentage of correct catheter placements compared to the modified Shukla group, suggesting that the Gupta method may lead to better positioning outcomes.

**Table 2:** Comparison of umbilical venous catheter tip position in the studied Gupta and modified Shukla’s groups as determined by X ray. *Significant.

Catheter position	Gupta group (N=88), n (%)	Modified Shukla Group (N=70), n (%)	X^2^ test value	P value
Correct position	55 (62.5)	32 (45.7)	7.62	0.02*
High position	20 (22.7)	15 (21.4)		
Low position	13 (14.8)	23 (32.9)		

An additional analysis was conducted to assess the impact of birth weight on the proper insertion of the UVC by comparing catheter tip positions between the Gupta and modified Shukla groups in infants with birth weights <1500 grams and ≥1500 grams. In neonates with a birth weight <1500 grams, the Gupta group had a significantly higher proportion of correct catheter positions (n=22, 62.9%) compared to the modified Shukla group (n=7, 38.8%), with a p-value of 0.02. However, for neonates with a birth weight ≥1500 grams, there was no significant difference between the Gupta group (n=33, 62.3%) and the modified Shukla group (n=25, 49.0%) in terms of correct catheter placement (p=0.18). Regarding high catheter positions, both groups had a similar proportion of placements in both birth weight categories, with no significant differences observed. The Gupta group had a higher proportion of low position, while in the ≥1500 gram category, the modified Shukla group had a higher proportion of low position (n=14, 27.5%) compared to the Gupta group (n=7, 13.2%). These differences, however, were not statistically significant in either birth weight category, as shown in Table 3.

**Table 3:** Comparison of the position of the umbilical venous catheter tip in birth weights <1500 and ≥ 1500 grams in the Gupta and modified Shukla groups, as determined by radiograph. *Bwt: Birth weight. **Significant.

Catheter position	Bwt* <1500 grams				Bwt ≥1500 grams			
	Gupta Group (N=35), n (%)	Modified Shukla group (N=19), n(%)	X^2^ test value	P value	Gupta Group (N=53), n (%)	Modified Shukla group (N=51), n (%)	X^2^ test value	P value
Correct position	22 (62.9)	7 (38.8)	5.92	0.05**	33 (62.3)	25 (49.0)	3.43	0.18
High position	7 (20.0)	3 (15.8)			13 (42.5	12 (23.5)		
Low position	6 (17.1)	9 (47.4)			7 (13.2)	14 (27.5)		

Table 4 presents the comparison of UVC tip positions by infant weight to gestational age category (AGA, SGA, and LGA) in the Gupta and modified Shukla groups, as determined by radiograph. In the AGA category, the proportion of correct catheter placements was higher in the Gupta group (n=32, 58.2%) compared to the modified Shukla group (n=23, 47.9%), though the difference was not statistically significant (p=0.20). However, in the SGA group, the Gupta group demonstrated a significantly higher proportion of correct placements (n=21, 70.0%) compared to the modified Shukla group (n=9, 42.9%), with a p-value of 0.04. In the LGA group, the correct placement rates were similar between the two groups, with 66.7% (n=2) in the Gupta group and no cases 0.0% in the modified Shukla group, but the difference was not statistically significant (p=0.50). In terms of low catheter positions, the modified Shukla group had a higher proportion of low placements in both the AGA (31.3% (n=15) vs. 16.4% (n=9)) and SGA (38.1% (n=8) vs. 13.3% (n=4)) categories, though these differences were not statistically significant for AGA (p=0.20), but significant for SGA group (p=0.04).

**Table 4:** Comparison of the position of the umbilical venous catheter tip by birth weight to gestational age category in the Gupta and modified Shukla groups, as determined by radiograph. ^a^Chi square test was used. ^b^Fisher exact was used. *Significant. AGA: appropriate for gestational age, SGA: small for gestational age, LGA: large for gestational age

Catheter position	AGA^a^ (n= 103)				SGA^b^ (n= 51)				LGA^b^ (n= 4)			
	Gupta (n= 55)	Shukla (n= 48)	Test value	P value	Gupta (n= 30)	Shukla (n= 21)	Test value	P value	Gupta (n= 3)	Shukla (n= 1)	Test value	P value
Correct	32 (58.2)	23 (47.9)	3.17	0.20	21 (70.0)	9 (42.9)	5.80	0.04*	2 (66.7)	0 (0.0)	0.45	0.50
High	14 (25.5)	10 (20.8)			5 (16.7)	4 (19.0)			1 (33.3)	1 (100.0)		
Low	9 (16.4)	15 (31.3)			4 (13.3)	8 (38.1)			0 (0.0)	0 (0.0)		

An analysis was conducted to compare the rate of catheter advancement, abnormal catheter tip position, and fix-on between the Gupta and modified Shukla groups. Regarding catheter advancement, the majority of advancements occurred during the first trial in both groups (80% (n=70) in the Gupta group and 82.9% (n=58) in the modified Shukla group), with no statistically significant difference between the groups (p=0.59). Concerning abnormal catheter tip position, a higher proportion of abnormal placements was observed in the modified Shukla group (n=23, 32.9%) compared to the Gupta group (n=13, 14.8%) with a statistically significant difference (p=0.02). The fix-on measurements showed no significant difference between the groups, with the Gupta group having a mean of 7.8±1.5 cm and the modified Shukla group that of 7.5±1.5 cm (p=0.45). The proportions of symmetrical and asymmetrical infant and catheter placements were similar between the groups, with no significant differences (p=0.12 for symmetrical and p=0.84 for asymmetrical).

An additional analysis was conducted to compare the rate of catheter advancement, abnormal catheter tip position, and fix-on between the Gupta and modified Shukla groups based on birth weights <1500 grams and ≥1500 grams. In both birth weight categories, the majority of catheter advancements occurred during the first trial, with no significant differences between the two groups (p=0.75 for <1500 grams and p = 0.71 for ≥1500 grams). Regarding abnormal catheter tip positions, a higher proportion of abnormal placements was observed in the modified Shukla group in the birth weight <1500 gram category (47.4% (n=9) vs. 17.1% (n=6)), and this difference was statistically significant (p=0.02). However, in the ≥1500 gram category, the difference between the groups was not statistically significant (p=0.18). The fix-on measurement, in terms of mean±SD, showed no significant difference between the groups for either birth weight category (p=0.98 for <1500 grams and p=0.55 for ≥1500 grams). The proportions of symmetrical and asymmetrical infants for catheter placements were similar between the groups, with no statistically significant differences in either birth weight category (p=0.99 and p=0.46 for <1500 grams, and p=0.23 and p= 0.52 for ≥1500 grams, respectively).

## Discussion

Umbilical vein catheterization is a crucial procedure in neonatal units, but it is associated with numerous complications [11,12]. Many of these complications stem from improper positioning of the catheter tips, whether too high or too low [6]. Morphometric measurements are more intuitive and less prone to the inaccuracies of weight-based formulas, especially in infants of varying body compositions [7]. In this study, we compared two methods: the Gupta method and the modified Shukla formula, which are formulas based on the distance from the base of the umbilicus to the nipple and the body weight of the infant, respectively. The position of the UVC tip was confirmed radiologically. Additionally, the study compared the catheter tip positions between the Gupta and modified Shukla groups in infants with birth weights <1500 and ≥1500 grams. The two studied groups were similar in terms of these critical neonatal parameters. Also, the reasons for hospitalization, including conditions like CPR, RDS, preterm birth, and extreme prematurity, were distributed similarly between the two groups, with no significant differences. This indicates that the clinical conditions leading to hospitalization were evenly balanced across both groups. The reasons for UVC were mainly for intravenous fluids and antibiotics, like those reported in other studies [13,14].

The results of this study revealed a significantly higher rate of correct UVC tip placements in the Gupta method (55 infants, 62.5%) compared to the modified Shukla method (32 infants, 45.7%) with a p-value of 0.02, suggesting that the Gupta method may be more effective for achieving proper catheter placement. Our findings, which show a 62.5% (55 infants) success rate for the Gupta method, are consistent with the superiority of this method as reported by Gupta et al. [7]. However, our success rate is lower than the 94% accuracy they reported. This difference may be attributable to variations in our study population or other factors (such as operator experience or measurement techniques). Nevertheless, the underlying principle of the Gupta method that morphometric measurements may be more reliable than weight-based formulas is supported by our results.

The Gupta method demonstrated a higher rate of correct UVC insertion in this study (55 infants, 62.5%) compared to other UVC methods. For instance, a prospective study conducted at the NICU of Al-Khansaa Maternity and Children's Teaching Hospital in Mosul, Iraq, included 97 neonates assigned to the Dunn’s group (49 infants) or the Shukla’s group (48 infants). The Dunn's method was more accurate than Shukla’s method for determining the optimal insertion length of UVCs (45% vs. 25%, p=0.04), especially in infants with birth weight <1500 grams (59% vs. 11%, p=0.003) [15]. A study by Verheij et al. in the Netherlands similarly found that 41% (28/69) of UVCs in the Dunn group were placed directly in the correct position, compared to 24% (20/84) in the Shukla group [6]. This reflects the poor accuracy of methods currently used to determine the umbilical catheter insertion length. These differences in correct placement rates could reflect variations in techniques, catheter insertion length, positioning guidelines, or procedural factors between the different methods.

In our stratified analysis by birth weight (<1500 grams and ≥1500 grams), significant differences in catheter tip positions were observed between the Gupta and Shukla groups. In neonates with birth weights less than 1500 grams, 62.9% (22 infants) of the Gupta group had correctly positioned catheters compared to 38.8% (seven infants) in the Shukla group. While not statistically significant, the rate of correct positions was also higher in the Gupta group for neonates with birth weights ≥1500 grams (33 infants, 62.3% versus 25 infants, 49%). Overall, the correct placement rates observed in previous studies were lower than those seen in our study for both groups. For example, in the Iraq study, the rate of correctly positioned UVC tips in infants with birth weights <1500 grams was significantly higher in Dunn's group (59%) compared to Shukla’s group (11%) [18]. Additionally, a randomized controlled trial by Sheta et al. in Canada compared Shukla’s formula with a surface measurement (SM) method based on the umbilicus-to-nipple distance. In this study, more extremely low birth weight (ELBW) infants in the SM group had correctly positioned UVC tips compared to those in the Shukla group (43.7% vs. 22.5%), which is consistent with the findings of our study. These results further support the potential advantages of the Gupta method in achieving more accurate UVC placements [16].

Stratified analysis by gestational age category revealed important differences in the performance of the Gupta and modified Shukla methods for UVC tip placement. In the appropriate for gestational age (AGA) group, the Gupta method showed a higher proportion of correct catheter placements (32 infants, 58.2%) compared to the modified Shukla method (23 infants, 47.9%) though this difference was not statistically significant (p=0.20). This suggests that, for AGA infants, both methods performed similarly in terms of achieving correct UVC placements, with Gupta showing a slight advantage in accuracy. In the small for gestational age (SGA) group, the Gupta method demonstrated a significantly higher proportion of correct placements (70.0%, 21 infants) compared to the modified Shukla method (42.9%, nine infants), with a p-value of 0.04. This suggests that the Gupta method may be particularly more effective for SGA infants, possibly due to better adaptation to their unique anatomical features, which require more precise catheter placement. Regarding low catheter placements, the modified Shukla method showed a higher proportion of low placements in both the AGA (31.3% (15 infants) vs. 16.4% (nine infants)) and SGA (38.1% (eight infants) vs. 13.3% (four infants)) categories. In the SGA group, the difference was statistically significant (p=0.04), suggesting that the modified Shukla method may be more likely to result in low catheter placements in SGA infants. Similarly, in a study of SGA patients (n=46) who had umbilical venous catheters placed using the modified Shukla-Ferrara formula, which mainly considers birth weight, a higher rate of low catheter positions was also observed [20]. In our study, the small sample size in the large for gestational age (LGA) group (n=4) limited the ability to draw conclusive results, making it difficult to definitively assess the relative performance of the two methods in this group.

In terms of catheter advancement, both of the studied groups showed a similar high rate of successful catheter advancement during the first trial, with 80.0% (70 infants) of cases in the Gupta group and 82.9% (58 infants) in the modified Shukla group. This indicates that both methods are equally effective in advancing the catheter, as there was no statistically significant difference between the two groups (p=0.59). The rate of catheter advancement also showed no significant difference between infants with birth weights <1500 grams and ≥1500 grams. However, when it comes to abnormal catheter tip positions, the modified Shukla group had a significantly higher rate of abnormal placements (23 infants, 32.9%) compared to the Gupta group (13 infants, 14.8%), with a p-value of 0.02. This trend was particularly evident in infants with birth weights <1500 grams. These findings suggest that the Gupta method may be more effective in achieving accurate catheter tip placement, thereby reducing the occurrence of abnormal placements. In the previously mentioned prospective study, the rate of abnormal umbilical venous catheter positioning ranged from 29% with the Dunn method to 37.5% with the modified Shukla method [15], which is higher than the rate observed in our study for the modified Shukla group.

When comparing the fix-on measurements, no significant difference was found between the Gupta and modified Shukla groups. Furthermore, stratification by birth weight also revealed no difference in insertion depth between the two methods. These results suggest that both methods are comparable in terms of catheter insertion depth, indicating that neither method leads to a significant variation in the fix-on measurement. This aligns with previous studies, which have shown that while methods may differ in placement accuracy or technique, the overall insertion depth tends to be relatively consistent across various approaches [15-18]. Also, the proportions of symmetrical and asymmetrical infants for catheter placements were similar between the Gupta and modified Shukla groups, with no statistically significant differences observed (p=0.12 for symmetrical and p=0.84 for asymmetrical infants). These findings align with the previous study results, which have shown that while different insertion techniques may affect overall catheter positioning, they do not necessarily result in significant differences in the symmetrical and asymmetrical infants [15].

Although UVC is a commonly performed, life-saving procedure, it carries the risk of serious complications that can lead to significant morbidity and mortality. Potential complications include infections, sepsis, as well as necrotizing enterocolitis [17]. Other complications include peritoneal extravasation and venous thrombosis [18,19]. In our study, no complications were recorded among the 158 neonates. This positive outcome was attributed to our proactive management protocol for UVC malpositioning. Specifically, when a device was found to be in a low position, it was promptly removed and reinserted to ensure correct placement. Conversely, if UVC was in a high position, it was carefully readjusted to the appropriate anatomical location. These immediate and targeted interventions effectively prevented complications associated with malpositioning. 

This study benefits from its randomized clinical trial design, which is a robust methodology for comparing the accuracy of two different formulas for estimating the UVC insertion depth. By randomly assigning neonates to either the umbilicus to nipple (UN) minus 1 cm formula or the modified Shukla birth weight-based formula, the study minimizes selection bias and ensures comparability between the groups. The study's detailed methodology, including well-defined inclusion and exclusion criteria and ethical considerations, enhances its credibility and reliability. To the best of our knowledge, this is the first randomized clinical trial in Saudi Arabia to compare the Gupta and modified Shukla methods using a relatively large sample size. Additionally, the study evaluates UVC placement accuracy across different birth weight and gestational age categories.

Despite its strengths, the study has several limitations. Although randomization was implemented, potential bias in procedure execution or inconsistencies in the application of the formulas may still have influenced the outcomes. Variations in technique and the experience of NICU physicians performing the UVC insertions could also lead to discrepancies in catheter placement. Additionally, the use of X-rays in this study may not provide the same level of detail or accuracy regarding soft tissues and structures as ultrasound. Lastly, the inability to utilize echocardiography or real-time ultrasonography, which are considered more reliable methods for precisely verifying the UVC tip placement, limited the study’s findings, as suggested by several authors [20,21].

## Conclusions

This study demonstrates that the Gupta method for UVC placement is associated with a higher rate of correct catheter tip positioning compared to the modified Shukla method, particularly in very low birth weight (LBW) and small for gestational age (SGA) infants. While both methods showed similar results in terms of catheter advancement, the Gupta method exhibited fewer abnormal catheter tip placements, suggesting better overall accuracy in positioning. Despite some limitations, such as the lack of real-time imaging techniques like echocardiography or ultrasound, this study provides valuable insights into UVC placement strategies and supports the use of the Gupta method for improved catheter positioning, particularly in neonates with lower birth weights. Further studies with larger sample sizes and the use of ultrasound for real-time imaging would help confirm these findings and assess long-term outcomes.

## References

[1] Cochran WD, Davis HT, Smith CA (1968). Advantages and complications of umbilical artery catheterization in the newborn. Pediatrics.

[2] Egan EA 2nd, Eitzman DV (1971). Umbilical vessel catheterization. Am J Dis Child.

[3] Gomella TL, Cunningham MD, Eyal FG, Zenk KE, 5th ed (2004). Neonatology, Management, Procedures, On-call Problems, Diseases and Drugs. New York: Lange Medical Books/McGraw-Hill.

[4] Kulkarni PB, Dorard RD (1979). Hydrothorax: a complication of intracardiac placement of umbilical venous catheter. J Pediatr.

[5] Dunn PM (1966). Localization of the umbilical catheter by post-mortem measurement. Arch Dis Child.

[6] Verheij GH, Te Pas AB, Witlox RS, Smits-Wintjens VE, Walther FJ, Lopriore E (2010). Poor accuracy of methods currently used to determine umbilical catheter insertion length. Int J Pediatr.

[7] Gupta AO, Peesay MR, Ramasethu J (2015). Simple measurements to place umbilical catheters using surface anatomy. J Perinatol.

[8] Mutlu M, Parıltan BK, Aslan Y, Eyüpoğlu İ, Kader Ş, Aktürk FA (2017). Comparison of methods and formulas used in umbilical venous catheter placement. Turk Pediatri Ars.

[9] Lean WL, Dawson JA, Davis PG, Theda C, Thio M (2019). Accuracy of five formulae to determine the insertion length of umbilical venous catheters. Arch Dis Child Fetal Neonatal Ed.

[10] Kieran EA, Laffan EE, O'Donnell CP (2016). Estimating umbilical catheter insertion depth in newborns using weight or body measurement: a randomised trial. Arch Dis Child Fetal Neonatal Ed.

[11] David N, Mangeret RM, Pfister RE (2017). Umbilical venous catheter-associated pleural effusions. Arch Dis Child Fetal Neonatal Ed.

[12] Mutlu M, Aslan Y, Kul S, Yılmaz G (2016). Umbilical venous catheter complications in newborns: a 6-year single-center experience. J Matern Fetal Neonatal Med.

[13] Gomella TL, Cunningham MD, Eyal FG, Tuttle DJ (2013). Neonatology: Management, Procedures, On-call Problems, Diseases, and Drugs. https://accesspediatrics.mhmedical.com/book.aspx?bookid=2762.

[14] Hoellering AB, Koorts PJ, Cartwright DW, Davies MW (2014). Determination of umbilical venous catheter tip position with radiograph. Pediatr Crit Care Med.

[15] Shareef AA, Mohammed RF, Kandla NA (2021). Assessment of umbilical venous catheter insertion depth using Dunn and Shukla method. Curr Pediatr Res.

[16] Sheta A, Kamaluddeen M, Soraisham AS (2020). Umbilical venous catheter insertion depth estimation using birth weight versus surface measurement formula: a randomized controlled trial. J Perinatol.

[17] Yeung CY (2020). Complications of umbilical venous catheters in neonates: a safety reappraisal. Pediatr Neonatol.

[18] D'Andrea V, Prontera G, Rubortone SA (2021). Umbilical venous catheter update: a narrative review including ultrasound and training. Front Pediatr.

[19] Goh SS, Kan SY, Bharadwaj S, Poon WB (2021). A review of umbilical venous catheter-related complications at a tertiary neonatal unit in Singapore. Singapore Med J.

[20] Sharma D, Farahbakhsh N, Tabatabaii SA (2019). Role of ultrasound for central catheter tip localization in neonates: a review of the current evidence. J Matern Fetal Neonatal Med.

[21] Ades A, Sable C, Cummings S, Cross R, Markle B, Martin G (2003). Echocardiographic evaluation of umbilical venous catheter placement. J Perinatol.

